# Effect of Phosphate Phase Incorporation on 3D-Printed Hydrogel Scaffolds: Towards Customizable Bone Graft Materials

**DOI:** 10.3390/gels11080665

**Published:** 2025-08-20

**Authors:** Andreea Trifan, Eduard Liciu, Andrei-Silviu Nedelcu, Mihai Dragomir, Doru-Daniel Cristea, Ciprian-Ștefan Mateescu, David-Andrei Nițulescu, Cătălina-Ana-Maria Cîrstea, Adela Banciu, Gabriela Toader, Aurel Diacon, Cristina Busuioc

**Affiliations:** 1 3D Printing Laboratory, Center of Innovation and e-Health, Carol Davila University of Medicine and Pharmacy, 020021 Bucharest, Romania; andreea.trifan@umfcd.ro (A.T.); mihai.dragomir@umfcd.ro (M.D.); doru.cristea@umfcd.ro (D.-D.C.); ciprian-stefan.mateescu@umfcd.ro (C.-Ș.M.); david-andrei.nitulescu2023@stud.umfcd.ro (D.-A.N.); catalina-ana-maria.cirstea2023@stud.umfcd.ro (C.-A.-M.C.); 2 Faculty of Chemical Engineering and Biotechnologies, National University of Science and Technology Politehnica Bucharest, 011061 Bucharest, Romania; 3 Faculty of Medical Engineering, National University of Science and Technology Politehnica Bucharest, 011061 Bucharest, Romania; andrei.nedelcu3110@stud.fim.upb.ro (A.-S.N.); adela.banciu@upb.ro (A.B.); 4 REOROM Laboratory, Hydraulics Department, Power Engineering Faculty, National University of Science and Technology Politehnica Bucharest, 060042 Bucharest, Romania; 5 Military Technical Academy, FERDINAND I, 050141 Bucharest, Romania; gabriela.toader@mta.ro (G.T.); aurel.diacon@mta.ro (A.D.)

**Keywords:** scaffolds, 3D printing, calcium phosphates, monetite, brushite, tissue engineering, hydrogels, biopolymers, composite materials

## Abstract

Bone defects remain a significant clinical challenge, creating a severe need for advanced biomaterials for tissue regeneration. This study addresses this issue by developing 3D-printed composite hydrogels containing alginate, gelatine, and resorbable calcium phosphates (monetite and brushite) for bone tissue engineering. The scaffolds were fabricated using extrusion-based 3D printing and evaluated for their morphology, porosity, mechanical strength, swelling, degradation, and in vitro mineralization, while their cytocompatibility was assessed using LIVE/DEAD cell viability assays. The key findings demonstrate that calcium phosphate incorporation enhanced the mechanical stability by 15–25% compared to the controls, and mineral deposition increased significantly in the composite scaffolds. The developed hydrogels are bioactive and represent promising, customizable scaffolds for bone regeneration. These results support their further investigation as viable alternatives to traditional bone grafts for clinical bone tissue engineering applications.

## 1. Introduction

The design and synthesis of advanced biomaterials with tailored chemical and structural properties are central to the progress of regenerative medicine [1] and tissue engineering. Hydrogels, in particular, have emerged as a versatile class of materials due to their tuneable physicochemical characteristics, high water content, and ability to mimic the extracellular matrix (ECM) of living tissues [2]. Natural polymers, such as alginate and gelatine, are widely used in hydrogel fabrication, offering abundant functional groups [3] for chemical modification, mild gelation conditions, and inherent biocompatibility [4]. Alginate, an anionic polysaccharide [5], forms hydrogels via ionic crosslinking with divalent cations, while gelatine, a denatured collagen derivative, provides cell-adhesive structures and improves the mechanical resilience of a network [6]. The combination of these polymers enables the creation of composite hydrogels with enhanced printability, elasticity, and biological performance, making them ideal candidates for the next generation of biomedical scaffolds [7].

The functional landscape of hydrogel-based biomaterials has been further expanded by the incorporation of inorganic phases [8], particularly calcium phosphate (CaP) minerals, such as monetite (CaHPO_4_) and brushite (CaHPO_4_·2H_2_O) [9]. These resorbable ceramics possess higher solubility than hydroxyapatite, allowing for controlled ion release and dynamic remodelling in physiological environments [10]. When integrated into polymeric hydrogels, calcium phosphates reinforce the mechanical framework and introduce bioactive sites for cell interaction, mineralization, and ion exchange [11]. Such organic–inorganic composites can be engineered to achieve a balance between elasticity, stability, and bioactivity, closely replicating the hierarchical structure of native tissues [2].

Recent advances in 3D printing [12], especially extrusion-based bioprinting, have revolutionized the fabrication of gel-based biomaterials. This technology enables precise spatial control over scaffold architecture [13] and composition, allowing for the creation of complex, patient-specific constructs [14] with interconnected porosity, tailored degradation rates, and spatial gradients in both their organic and inorganic components [15]. The ability to systematically modulate parameters, such as polymer concentration, mineral content, crosslinking density, and printing conditions, is essential for optimizing the physicochemical and functional properties of the final scaffolds [16].

A critical challenge in the design of hydrogel-based biomaterials is understanding and controlling the interplay between the chemistry of the polymer matrix, the nature and distribution of the inorganic phase [17], and the resulting structure–property relationships. The swelling behaviour, degradation kinetics, and mechanical performance are governed by crosslinking mechanisms, polymer chain interactions, and the presence of mineral fillers [18]. The solubility and morphology of CaP phases—monetite versus brushite—directly influence the scaffold resorption profile and ion release, which are crucial for applications requiring dynamic integration with living tissues [9].

The clinical relevance of these materials is underscored by the global burden of bone defects. According to the World Health Organization, approximately 440 million people suffer bone fractures annually, resulting in over 26 million years of life lived with disability worldwide [19]. More than 2 million bone graft procedures are performed each year, making bone the second-most transplanted tissue after blood [18]. However, current solutions, such as autografts and allografts, are limited by donor site morbidity, tissue availability, and risks of disease transmission or immune rejection, highlighting the urgent need for alternative biomaterials that can support tissue regeneration without the drawbacks of traditional grafts [16].

The past several years have seen remarkable progress in the field of gel-based biomaterials [20] and 3D-printed scaffolds, with numerous studies demonstrating their potential for clinical translation [21]. For example, a 2025 study [22] evaluated the osteogenic potential of 3D-printed scaffolds composed of allograft bone, alginate, and gelatine, functionalized with stromal vascular fraction (SVF) and platelet-rich fibrin (PRF). In animal models, these implants showed significantly enhanced bone formation and angiogenesis, as evidenced by a histological analysis and micro-CT imaging, compared to scaffolds lacking SVF/PRF. This case highlights the effectiveness of combining natural matrix components with autologous growth factors for stimulating regeneration and underscores the importance of multi-component compositions and the controlled delivery of bioactive agents [23].

Another recent investigation focused on 3D-printed alginate–gelatine hydrogels with varying concentrations of calcium phosphate for bone regeneration [11], evaluating mesenchymal stem cell proliferation and osteogenic differentiation. The results demonstrated that a moderate monetite content (1–6%) achieved a balance between extrudability, porosity, and controlled Ca^2+^/[PO_4_]^3−^ ion release, stimulating osteoblast markers more effectively than scaffolds with no or excessive CaP [11].

Hybrid scaffolds incorporating calcium-doped bioglass and polymers, fabricated by direct ink writing [12], have shown excellent synergy between the mechanical properties and surface bioactivity. In femoral defect models in mice, these scaffolds increased the rate of bone formation and mineralization compared to the controls, as demonstrated by micro-CT and histological analysis [24]. These findings highlight the importance of combining ion-releasing mineral phases with cell-supportive polymers for enhanced osteogenesis [24,25].

Advanced printing strategies now enable the fabrication of scaffolds with graded porosity [26]. inspired by the hierarchical architecture of bone [27]. For example, hydrogel-based scaffolds with macro- and micro-porosity gradients, achieved through variable printing parameters, have been shown to promote differential cell migration and enhanced bioactivity, particularly when loaded with calcium phosphates [28]. In vivo, these scaffolds have supported improved bone growth in regions with optimized porosity gradients.

Across these case studies, the importance of standardizing the printing parameters—such as the hydrogel viscosity [29], gelation time, nozzle diameter, pressure, and temperature—and integrating real-time feedback from bioink behaviour into computer-aided design (CAD) simulations [30] has become increasingly clear for ensuring reproducibility and clinical traceability [31]. Moreover, the current research emphasizes compliance with regulatory standards and pharmacovigilance, particularly with respect to the United States Food and Drug Administration (FDA) or the European Medicines Agency (EMA) guidelines for bioprinting materials and procedures [32].

In this study, we report the development and comprehensive characterization of 3D-printed composite hydrogels based on alginate, gelatine, and resorbable calcium phosphates. By systematically varying the composition and processing parameters, we aim to elucidate how the chemical and structural features of these materials dictate their swelling behaviour, degradation, mechanical properties, and potential for further functionalization. These insights are pivotal for advancing the rational design of next-generation gel-based biomaterials for a wide range of biomedical applications, including but not limited to bone tissue engineering.

## 2. Results and Discussion

### 2.1. CaP Powders Characterization

#### 2.1.1. X-Ray Diffraction

**Figure 1:**
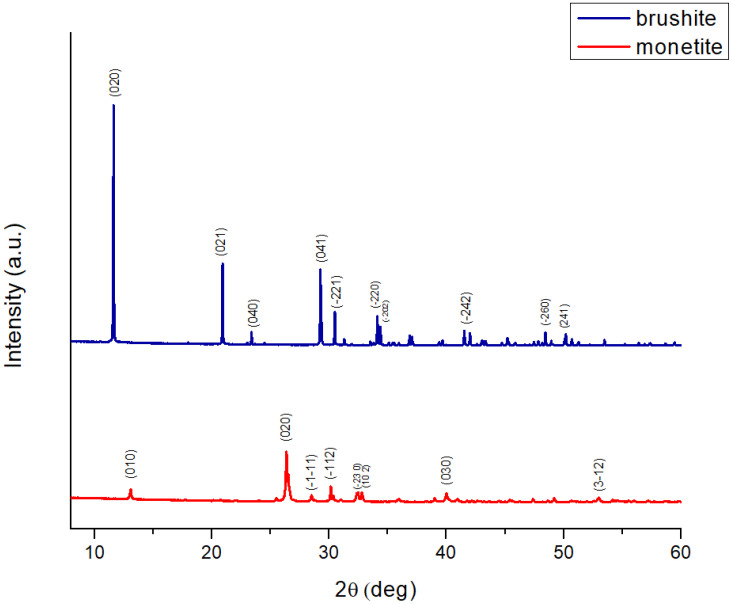
XRD patterns of the brushite and monetite powders.

The X-ray diffraction (XRD) pattern of brushite (Figure 1) was analysed using the ICDD reference card 00-009-0077. The diffractogram displays characteristic brushite peaks, with the most intense reflection at approximately 11.6° (2*θ*), corresponding to the (020) crystallographic plane. This peak is the clearest signature of brushite, given its very large interplanar spacing (~7.6 Å), which indicates well-developed crystals with preferred orientation. Other significant peaks include reflections at (021) ~20.9°, (040) ~23.3°, (041) ~27.0°, and (220) ~31.1°. The reflections are well separated and show no overlap, indicating high phase purity.

The XRD pattern of monetite (Figure 1) was analysed using the ICDD reference card 00-009-0080. The monetite diffractogram lacks reflections in the low-angle region (below 11–12°), which excludes the presence of brushite. The dominant reflection is observed at approximately 26.5–27.0° (020), accompanied by clear peaks at (010) ~12.3°, (−11) ~29.1°, (−112) ~30.0°, (230) ~32.1°, (120) ~34.2°, (030) ~40.5°, and (3–2) ~53.2°. These peaks are relatively broad and of medium intensity, suggesting moderate crystallinity, which is typical for monetite obtained by the thermal conversion of brushite (~100 °C, 24 h) or by coprecipitation synthesis at a low pH (acidic, <4.5) [33].

#### 2.1.2. Fourier Transform Infrared Spectroscopy

**Figure 2:**
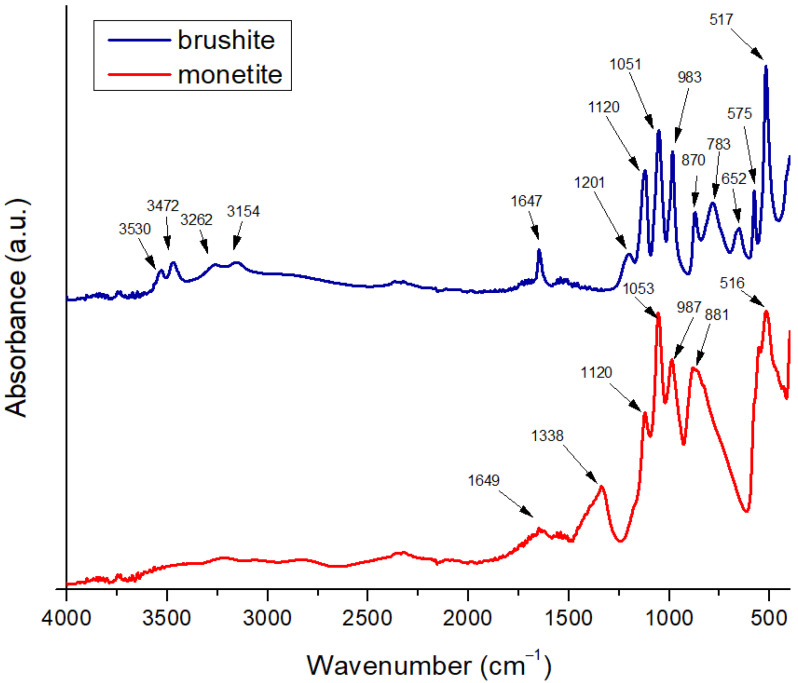
FTIR spectra of the brushite and monetite powders.

The Fourier transform infrared spectroscopy (FTIR) for brushite (Figure 2) exhibits a broad vibrational band corresponding to O–H stretching in the approximate range of 3600–3000 cm^−1^, with local maxima near 3530, 3472, 3262, and 3154 cm^−1^. These features confirm the presence of crystallization water within the dihydrate structure of CaHPO_4_·2H_2_O. This complex band arises from vibrations of hydroxyl groups bound within the crystalline lattice and intercalated water molecules, which absorb at slightly different energies due to their chemical environment and hydrogen bonding [34]. A pronounced peak at around 1647 cm^−1^ is attributed to the bending vibrational mode of water (*δ*–O–H), clearly indicating the bending vibration of crystallized water molecules and serving as a characteristic signature of brushite. The presence of this bending signal at approximately 1647 cm^−1^ confirms the dihydrate nature of the phase and distinguishes brushite from anhydrous calcium phosphate forms [35].

In the phosphate group region, the brushite spectrum shows a P–O stretching band near 1201 cm^−1^ and another intense peak around 1120 cm^−1^, reflecting the stretching vibrations of [PO_4_]^3−^ groups in the hydrated structure. These P–O stretching bands are slightly shifted compared to those of monetite, due to interactions with the crystallization water and local geometric changes within the lattice. In the fingerprint region (<1000 cm^−1^), distinct maxima appear at 983, 870, 783, 652, 575, and 517 cm^−1^, corresponding to various vibrational modes of O–P–O and P–O in the hydrated environment, as well as possible rotational modes of bound water molecules (*ρ*-H_2_O). For example, the band at 783 cm^−1^ can be assigned to symmetric vibrations of P–O–H groups, while those at 652 and 575 cm^−1^ correspond to bending modes of O–P–O groups in the phosphate tetrahedron, influenced by the structural water presence. The intense peak at 517 cm^−1^ is associated with rotational or mixed vibrations involving [PO_4_]^3−^ groups and crystallization water, typical of well-crystallized brushite [36].

The relative width of the O–H stretching bands and the bending band at 1647 cm^−1^ indicate a moderate degree of crystallinity, in which the crystallization water is well integrated but exhibits local variability, generating multiple absorption components in the 3600–3000 cm^−1^ region. The absence of unusual additional signals suggests the purity of the brushite phase in the powder, without major impurities such as carbonate (which would generate bands near 1420–1450 cm^−1^). Overall, the spectrum confirms the dihydrate structure: characteristic broad O–H stretching bands, clear H–O–H bending vibrations at 1647 cm^−1^, specific P–O stretching bands at 1201 and 1120 cm^−1^, and multiple fingerprint bands [37].

On the other hand, the FTIR spectrum of monetite is characterized by the absence of intense vibrational bands attributed to O–H stretching in the 3600–3000 cm^−1^ region, confirming the anhydrous nature of CaHPO_4_ and the lack of crystallization water. Any weak signals in this region may arise from superficially adsorbed water but do not produce a broad, intense band as observed in brushite. A modest peak near 1649 cm^−1^ may correspond to bending vibrations of residual H–O–H molecules; however, its low intensity confirms that no significant structural water is present in monetite [35].

The phosphate stretching region of monetite features strong peaks around 1053 and 1120 cm^−1^, indicative of symmetric [PO_4_]^3−^ vibrations within an anhydrous environment. The peak at 1120 cm^−1^ may reflect slight structural variations or local phosphate interactions, though the main reference band for monetite is typically reported near 1081–1053 cm^−1^ in the literature. In the fingerprint region, significant peaks appear at 987 and 881 cm^−1^, assigned to vibrational modes of O–P–O and P–O–H within the anhydrous phosphate tetrahedron, as well as a peak at 516 cm^−1^ associated with bending vibrations of O–P–O groups in the absence of crystallization water. Additionally, a signal at 1338 cm^−1^ suggests possible traces of carbonate within the lattice or minor impurities, attributed to vibrations of CO_3_^2−^ groups, which may arise from CO_2_ adsorption during handling and can influence the material’s solubility [36].

Compared to brushite, the monetite phosphate bands are narrower and better defined in the phosphate-specific region, indicating a well-ordered anhydrous crystalline structure. The absence of O–H stretching and H–O–H bending bands characteristic of brushite, combined with the pronounced P–O stretching bands near 1053 and 1120 cm^−1^ and the fingerprint bands at 987, 881, and 516 cm^−1^, confirm the identity of monetite [36].

#### 2.1.3. Scanning Electron Microscopy and X-Ray Energy-Dispersive Spectroscopy

**Figure 3:**
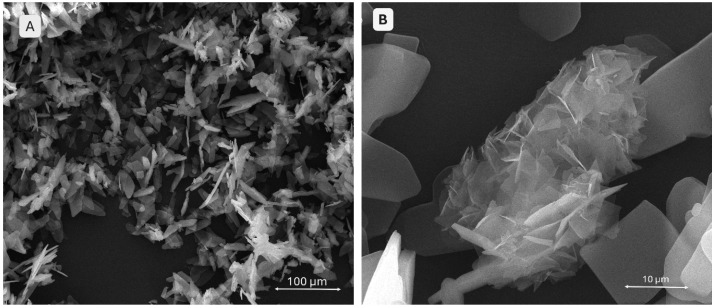
SEM images of the brushite powder (**A**,**B**) at different magnifications.

A scanning electron microscopy (SEM) analysis was conducted on both the monetite and brushite powders, as well as on the 3D-printed scaffolds. The images in Figure 3 provide detailed information on the morphology of the brushite. In image A, taken at a lower magnification compared to image B, the particles are arranged chaotically without a well-defined orientation. The particles exhibit elongated, acicular, or foil-like shapes characteristic of brushite. Image B, at a higher magnification, reveals very thin platelet-like structures with well-defined, slightly rounded edges, while the centre shows an agglomerate of such platelets, giving the material a lamellar appearance.

**Figure 4:**
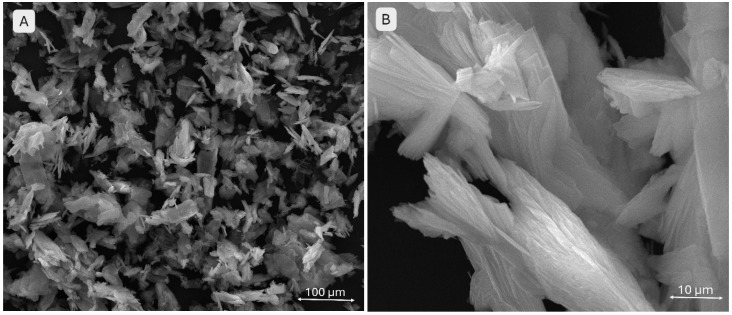
SEM images of the monetite powder (**A**,**B**) at different magnifications.

Figure 4 illustrates the morphology of the monetite. Image A provides an overview of the sample, showing particle agglomerates rather than isolated particles, with a relatively uniform size distribution. The structures maintain their lamellar morphology. Image B highlights the same platelet shapes but arranged in an ordered, stratified fashion with sharper edges, resembling “ice flakes.”

**Figure 5:**
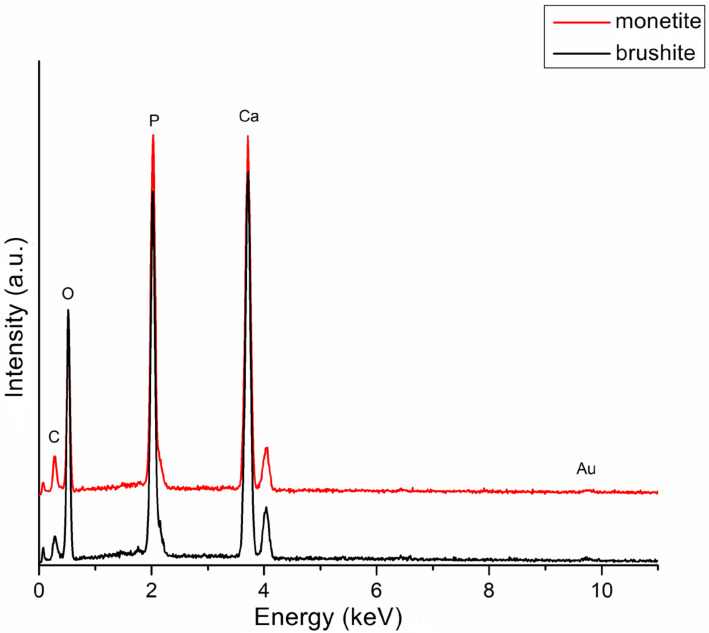
EDS spectra of the brushite and monetite powders.

Energy-dispersive X-ray spectroscopy (EDS) performed on the monetite and brushite powders (Figure 5) revealed spectral lines associated with the following elements: calcium (Ca), phosphorus (P), carbon (C), oxygen (O), and gold (Au). Several observations can be made from Figure 5 regarding the two samples: (i) the most prominent peaks correspond to phosphorus and calcium in both powders, indicating successful synthesis with a consistent calcium phosphate composition and similar signal intensities; (ii) the oxygen peak is higher in the brushite, confirming the presence of water in its composition. The carbon signal, considerably smaller than the others, arises from the carbon tape used to fix the samples on the aluminium substrate, while the gold signal is due to the coating applied to improve image stability, as the samples are non-conductive.

### 2.2. Composite Hydrogel Characterization

#### 2.2.1. Rheological Evaluation

Among the six compositions, only two were selected for rheological evaluation. A formulation similar to the S1–S3 compositions, containing 8% gelatine and 7% alginate, was characterized in our previous work [38]. Furthermore, this study focused on investigating the effects of CaP incorporation on the rheological properties of printable hydrogels. Specifically, we analysed the polymer-only formulation containing 12% gelatine, 5% alginate, and 1% CMC, alongside the composite with an additional 3% monetite, to evaluate how the inclusion of the inorganic phosphate phase influenced the material behaviour.

**Figure 6:**
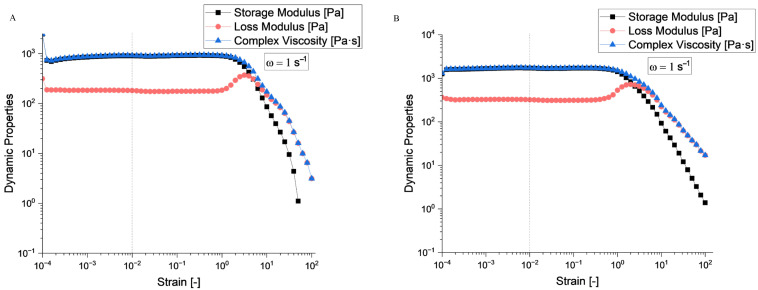
Amplitude sweep evaluation for (**A**) S4 and (**B**) S5 hydrogel compositions.

**Figure 7:**
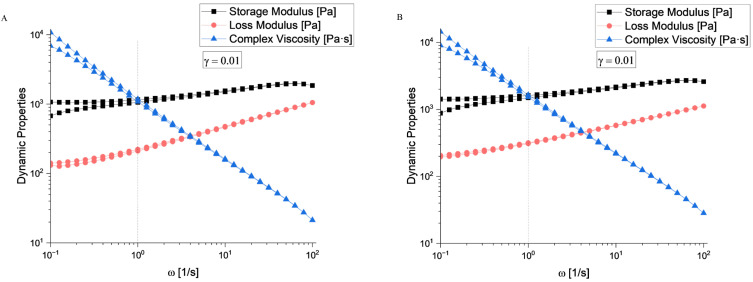
Frequency sweep evaluation for (**A**) S4 and (**B**) S5 hydrogel compositions.

According to the rheological evaluation (Figure 6A and Figure 7A), sample S4 exhibited a well-defined linear viscoelastic plateau extending from approximately 10^−4^ to 10^−1^ strain units, where both the storage modulus (*G*′) and loss modulus (*G*″) remained constant at approximately 10^3^ Pa. The critical strain marking the end of the linear viscoelastic region (LVR) occurred around *γ* = 0.1, beyond which both moduli began to decrease significantly.

Sample S5 (Figure 6B and Figure 7B) demonstrated similar behaviour with a slightly more robust structure, maintaining linearity up to comparable strain levels. The storage modulus values were consistent with those of S4, indicating similar elastic properties in the undeformed state.

Beyond the LVR, both samples exhibited structural breakdown characterized by a rapid decrease in both the *G*′ and *G*″. This behaviour is typical of structured fluids, where increasing the deformation amplitude disrupts the internal network structure. The crossover point where *G*′ = *G*″ occurred at high strain values (around 10^1^), indicating a transition from predominantly elastic to viscous behaviour.

Sample S4 showed a crossover frequency at approximately 1 rad/s, where *G*′ = *G*″. At low frequencies (long time scales), the loss modulus dominates (*G*″ > *G*′), indicating liquid-like behaviour. At higher frequencies (short time scales), the storage modulus becomes dominant (*G*′ > *G*″), reflecting solid-like behaviour. Sample S5 exhibited a similar crossover pattern, but with the intersection occurring at a slightly different frequency, suggesting variations in the characteristic relaxation time between the two samples.

The complex viscosity (η*) for both samples demonstrated typical shear-thinning behaviour across the frequency range. Based on the rheological fingerprints, both samples can be classified as viscoelastic materials with gel-like characteristics. The presence of well-defined LVR, frequency-dependent crossover behaviour, and structured breakdown patterns are consistent with materials possessing a weak gel structure that can be disrupted by moderate deformation, thixotropic potential suggested by the strain-dependent structural breakdown, and intermediate viscoelastic properties between purely elastic solids and viscous liquids.

**Figure 8:**
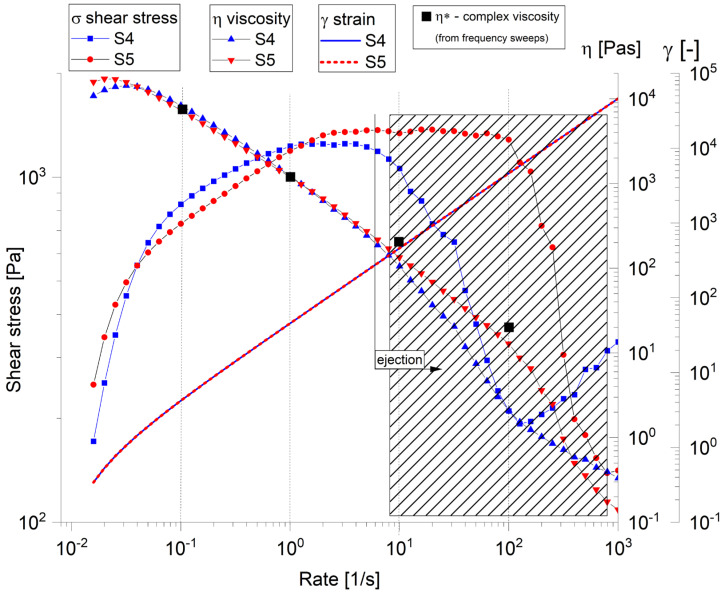
Flow curve of tested samples.

Both samples exhibited typical shear-thinning behaviour, wherein the viscosity decreased with an increasing shear rate, which is typical for structured or polymeric materials. The viscosity profiles of the two samples closely overlapped, as shown in Figure 8, indicating similar rheological properties. At low-to-moderate shear rates, the materials maintained their structural integrity, as evidenced by the high and relatively stable viscosity and shear stress values. However, beyond a critical shear rate of approximately 100 s^−1^, the data became increasingly scattered. This sudden decli7ne corresponds directly to the phenomenon of “ejection from the gap,” during which the sample was expelled from between the rheometer plates at elevated shear rates, resulting in unreliable and artificially reduced measurements. Under the influence of strong centrifugal forces generated during rotation, localized dense regions or aggregates tended to detach from the bulk and were physically thrown outward, particularly at the edges of the plates. These expelled fragments compromised the sample’s integrity within the measurement gap, causing abrupt changes in the shear stress at high shear rates and ultimately affecting the accuracy of the rheological assessment in that region [39]. Both samples S4 and S5 demonstrated remarkably similar rheological signatures, suggesting comparable material compositions and structures, despite the addition of CaP powder in S5.

#### 2.2.2. Filament Collapse Testing

**Figure 9:**
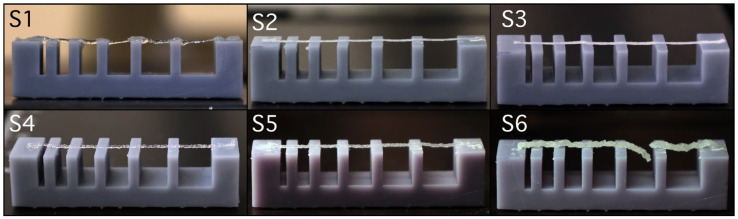
Filament collapse test for the (**S1**–**S6**) hydrogel compositions.

Comparative analysis of images captured during 3D printing (Figure 9) highlights the filament behaviour of sample S6, composed of 12% gelatine, 5% alginate, 1% CMC, and 3% brushite, which exhibited fracture when spanning gaps of 4 and 5 mm between supporting pillars. This instability is associated with its high viscosity and dense paste-like behaviour, despite using a larger 22G nozzle. In contrast, samples S2 and S3, both containing 8% gelatine, 7% alginate, 1% CMC, and 5% calcium phosphate (monetite in S2, brushite in S3), extruded continuously and uniformly, demonstrating that a more fluid polymeric base supports uninterrupted flow.

Sample S1, consisting of 8% gelatine, 7% alginate, and 1% CMC, without calcium phosphate, produced stable filaments, albeit with minor heterogeneities attributed to air bubble inclusion during preparation. For samples S4 and S5, both with 12% gelatine and 5% alginate, differences arose from the addition of 3% monetite in S5, which did not significantly disrupt the filament continuity compared to S4, suggesting that monetite up to this concentration is compatible with network stability. Compared to the monetite in S5, the brushite in S6 exhibited a more pronounced destabilizing effect, underscoring the importance of CaP type in selecting the extrusion parameters.

#### 2.2.3. Extrusion-Based 3D Bioprinting

**Figure 10:**
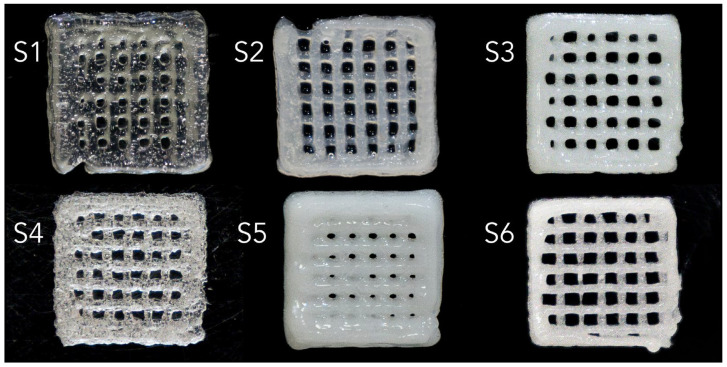
Visual aspect of the 3D-printed scaffolds, using the (**S1**–**S6**) hydrogel compositions.

Six scaffolds were obtained via extrusion-based bioprinting, as shown in Figure 10, with different hydrogel compositions and different degrees of translucidity, based on the amount of CaP powders introduced. The Pristine polymeric matrices S1 and S4 are characterized by the presence of air bubbles in the bioprinter cartridge, while S2–S3 and S5–S6 illustrate a homogenous dispersion of the composite phase, with varying degrees of stability, as shown from the shape of the pores.

#### 2.2.4. Fourier Transform Infrared Spectroscopy

**Figure 11:**
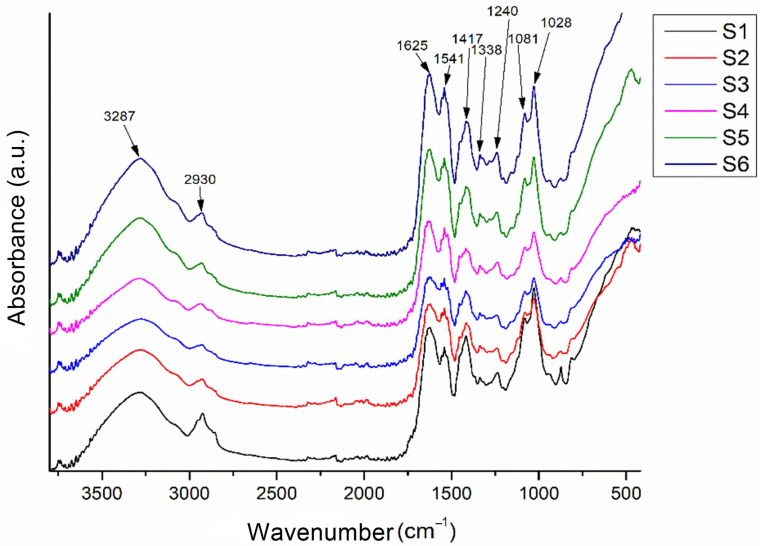
FTIR spectra of the 3D-printed scaffolds S1–S6.

FTIR spectra of composite scaffolds based on gelatine, alginate, and CMC loaded with CaP (monetite, brushite) exhibit characteristic bands attributable to the polymeric and mineral components, as shown in Figure 11. In the 3600–3000 cm^−1^ region, O–H and N–H stretching vibrations are observed, associated with gelatine, alginate, and CMC, as well as the crystallization water in the brushite. The 1750–1500 cm^−1^ region contains bands corresponding to amide I and II from the proteinaceous gelatine, which become more pronounced with an increasing gelatine content. Between 1616 and 1419 cm^−1^, asymmetric and symmetric COO^−^ bands from the alginate confirm polysaccharide integration, while the CMC contributes C–O–C and C–O vibrations in the 1300–1000 cm^−1^ range. The introduction of monetite and brushite generates additional bands specific to P–O bonds in the 1130–1030 cm^−1^ region (P–O stretching vibrations) and fingerprint bands below 800 cm^−1^ (associated with P–O–P and P–O(H) vibrations). The differences between monetite and brushite are reflected in the width and position of phosphate bands due to the presence of crystallization water in brushite and its distinct crystal structure.

#### 2.2.5. Scanning Electron Microscopy and X-Ray Energy-Dispersive Spectroscopy

**Figure 12:**
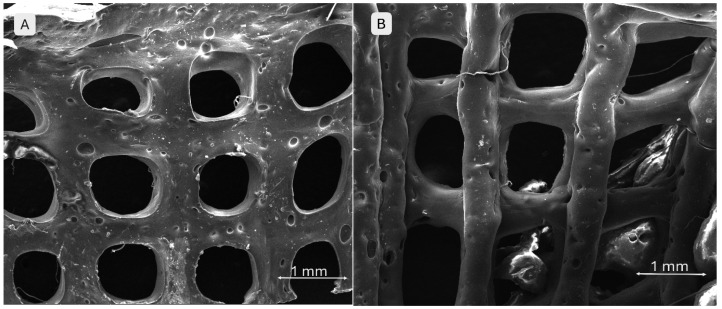
SEM images of the 3D-printed pristine scaffolds: (**A**) S1, (**B**) S4.

The SEM images in Figure 12 demonstrate well-defined filament and pore morphologies for the polymeric compositions without phosphate additives. In image A (S1), thin walls, clearly delineated pore edges, and an aerated architecture with an open porous network are visible, suggesting good printability. Conversely, image B (S4) shows a more compact structure with thickened, slightly collapsed walls, indicating higher viscosity of the mixture and improved geometric fidelity.

**Figure 13:**
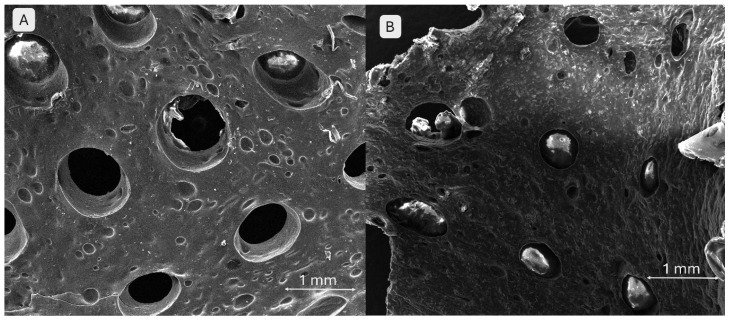
SEM images of the 3D-printed monetite-containing scaffolds: (**A**) S5—3% monetite; (**B**) S2—5% monetite.

The addition of monetite significantly affects the scaffold architecture (Figure 13). Image A (3% monetite) shows well-defined pores with slightly irregular edges and a surface exhibiting fine but uneven mineral particle agglomerations, suggesting the partial integration of monetite within the hydrogel matrix. Image B (5% monetite) reveals a denser morphology with a visibly reduced pore size. The pore edges appear deformed, likely due to the increased mixture rigidity. The higher monetite content seems to enhance the structural stability but at the expense of printing fidelity. The mineral particles are more prominent, potentially favouring scaffold osteoconductivity.

**Figure 14:**
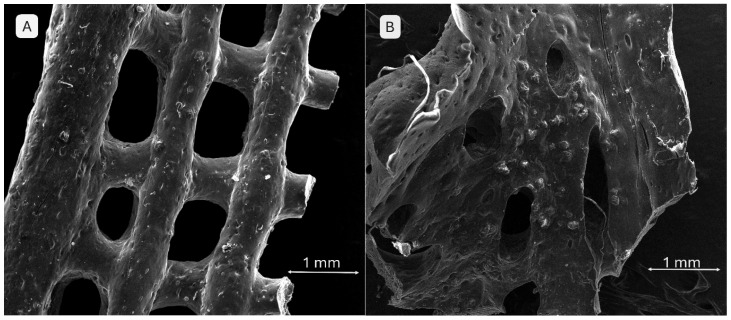
SEM images of the 3D-printed brushite-containing scaffolds: (**A**) S6—3% brushite; (**B**) S3—5% brushite.

The brushite-containing scaffolds (Figure 14) exhibit behaviour distinct from those containing monetite. In image A (3% brushite), the network structure is well preserved, displaying a clear and relatively uniform porous architecture. The surface appears smoother, and the brushite particles are less conspicuous at this concentration, suggesting a uniform distribution and weaker interaction with the polymer matrix. In image B (5% brushite), an irregular morphology is observed, characterized by asymmetric pores and a partial collapse of the structure in certain areas. The increased concentration of brushite appears to negatively affect the structural fidelity and printing homogeneity. This composition exhibits an apparently lower porosity and a more fragile structure, possibly due to the weak interactions between the mineral phase and the polymer network.

**Figure 15:**
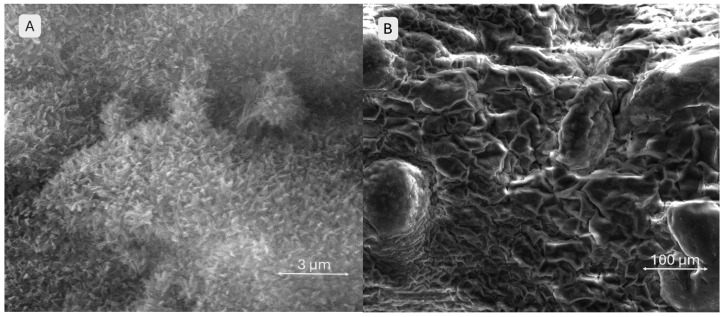
SEM images of the 3D-printed pristine scaffold S1 surface after 28 days of SBF immersion, (**A**,**B**) at different magnifications.

In Figure 15, image A shows a surface characterized by numerous submicron filamentous protrusions, resembling a carpet of fine, grass-like fibres. This fibrillar texture indicates the predominance of the polymeric network (gelatine, alginate, and CMC) and its interaction with the simulated body fluid (SBF) environment. Image B depicts the same scaffold at a larger scale, highlighting the clear contours of macroporous channels and pores. Since the material consists solely of alginate, gelatine, and CMC, the surface exhibits a slightly wavy texture with minor irregularities caused by polymer contraction and relaxation during drying, as well as its interaction with the SBF.

**Figure 16:**
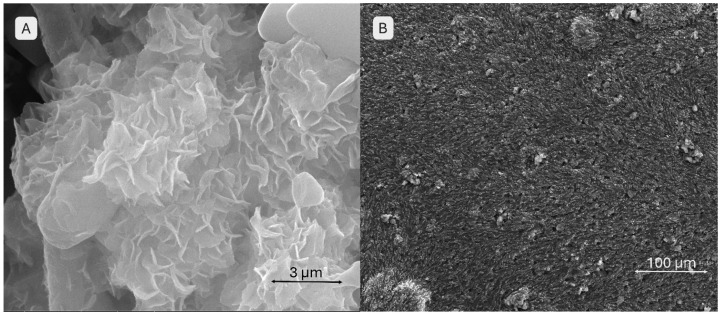
SEM images of the 3D-printed brushite-containing scaffold S3 surface after 28 days of SBF immersion: (**A**,**B**) at different magnifications.

The SEM images (Figure 16) provide a close-up view (image A) of the brushite-containing scaffold, where the surface appears as an assembly of thin, wavy, overlapping sheets resembling flower petals. This stratified and complex morphology suggests the presence and reactivity of mineral particles in contact with the SBF, with the brushite maintaining its initially fragmented structure. The high density of these sheets indicates enhanced bioactivity. Image B shows a surface densely covered by mineral particles arranged in a relatively ordered network, without obvious large pores. At this scale, the aggregates overlap in an apparently organized manner. The particles appear uniformly distributed, forming a slightly irregular but consistent relief across the entire surface, suggesting homogeneous mineral deposition.

**Figure 17:**
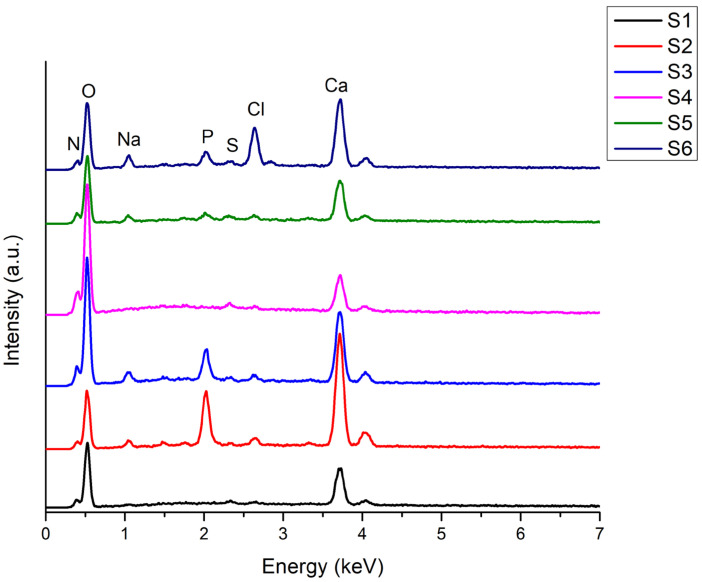
EDS spectra of the 3D-printed scaffolds S1–S6.

The EDS analysis of the 3D-printed scaffolds (Figure 17) reveals the presence of several elements: calcium (Ca), phosphorus (P), oxygen (O), nitrogen (N), sodium (Na), sulphur (S), and chlorine (Cl). The strong oxygen peak in all the samples reflects the hydrogel’s composition (gelatine and alginate), with both polymers containing oxygenated groups (hydroxyl, carbonyl). In the mineral-containing samples, oxygen also originates from the phosphate groups [PO_4_]^3−^ and, in the case of brushite, from crystallization water. Nitrogen is specific to the amidic bonds from gelatine, confirming its incorporation. The nitrogen signal intensity is slightly increased in the samples with 12% gelatine (S4–S6) compared to those with 8% gelatine (S1–S3), reflecting a higher proportion of amino groups. The phosphorus peak is absent in the samples without calcium phosphate (S1, S4) and present in samples containing monetite and brushite, as expected. The intensity of the phosphorus signal correlates with the CaP content: the samples with 5% CaP (S2, S3) show stronger signals than those with 3% CaP (S5, S6), confirming mineral phase incorporation. Chlorine appears in the spectra due to the use of the crosslinking solution, an aqueous CaCl_2_ solution; Cl^−^ ions may remain in the network after gelation and incomplete washing. The intensity of the chlorine signal can be higher immediately after crosslinking and decreases after multiple washing steps. Comparing the samples, a weak chlorine signal suggests efficient washing, while a stronger signal may indicate the retention of Cl^−^ ions within the gel structure. The calcium peak confirms the presence of the CaP phase in samples S2, S3, S5, and S6. Its intensity reflects the CaP percentage: S2 and S3 (5% CaP) exhibit a higher intensity, while S5 and S6 (3% CaP) show a lower intensity. In the samples without CaP (S1, S4), the calcium originates from CaCl_2_ crosslinking. The sodium peak derives from the sodium alginate used in the hydrogel composition.

#### 2.2.6. Printing Accuracy

**Figure 18:**
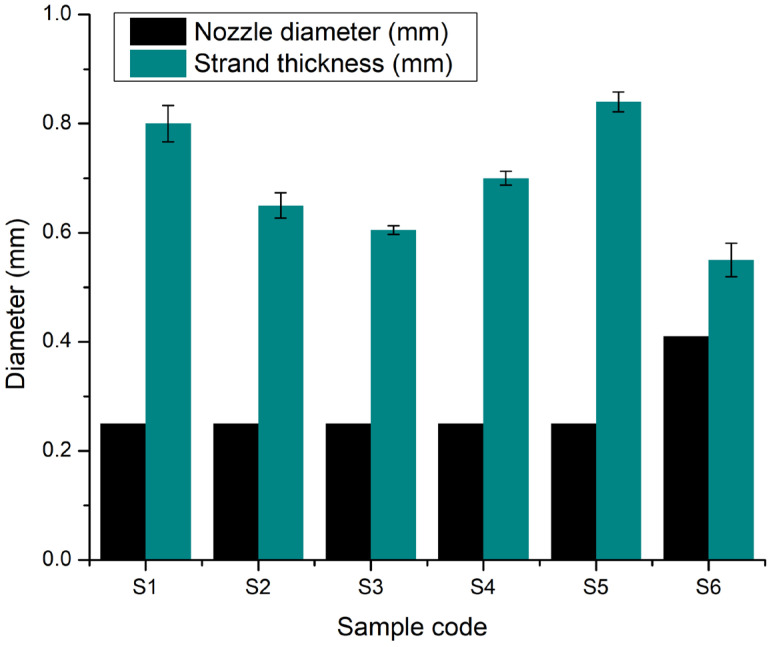
Comparative graph of the printing accuracy via strand thickness measurements and nozzle diameter used for the 3D-printed scaffolds S1–S6. The data are presented as the mean ± standard deviation (*n* = 3 per group).

To highlight how the composition influences the geometric characteristics of the extruded filaments, the comparison of filament diameters presented in Figure 18 provides valuable insights into the role of each component. The filaments produced by samples S1 (8% gelatine, 7% alginate, and 1% CMC) and S2 (8% gelatine, 7% alginate, 1% CMC, and 5% monetite) exhibited similar diameters around 0.80–0.85 mm, indicating that the addition of monetite did not significantly alter the non-Newtonian behaviour of the base hydrogel. In contrast, sample S3 (8% gelatine, 7% alginate, 1% CMC, and 5% brushite) showed a slightly reduced expansion, with filament diameters close to 0.70 mm, suggesting that brushite, due to its distinct crystalline structure, imparts additional rigidity to the network, limiting radial swelling.

Turning to the samples with increased gelatine content (12%), S4 (12% gelatine, 5% alginate, and 1% CMC) produced filaments approximately 0.75 mm in diameter, indicating that a higher gelatine proportion promotes increased viscosity and slightly reduces filament expansion. The addition of monetite in S5 (12% gelatine, 5% alginate, 1% CMC, and 3% monetite) resulted in an average filament diameter of about 0.85 mm, similar to that of S1 and S2, demonstrating that a moderate monetite content does not counteract gelatine’s effect on filament swelling. Conversely, sample S6 (12% gelatine, 5% alginate, 1% CMC, and 3% brushite), printed with a 22G nozzle, exhibited a filament diameter of approximately 0.55 mm; this outcome reflects both the influence of the larger nozzle and the more pronounced stiffening effect of brushite.

Overall, these comparisons reveal two distinct trends: the gelatine content and the presence of CMC significantly increased filament swelling by raising the hydrogel viscosity, while the type and concentration of calcium phosphate (monetite versus brushite) influenced network rigidity, with the monetite exerting a milder effect on swelling compared to brushite.

#### 2.2.7. Porosity Evaluation

**Figure 19:**
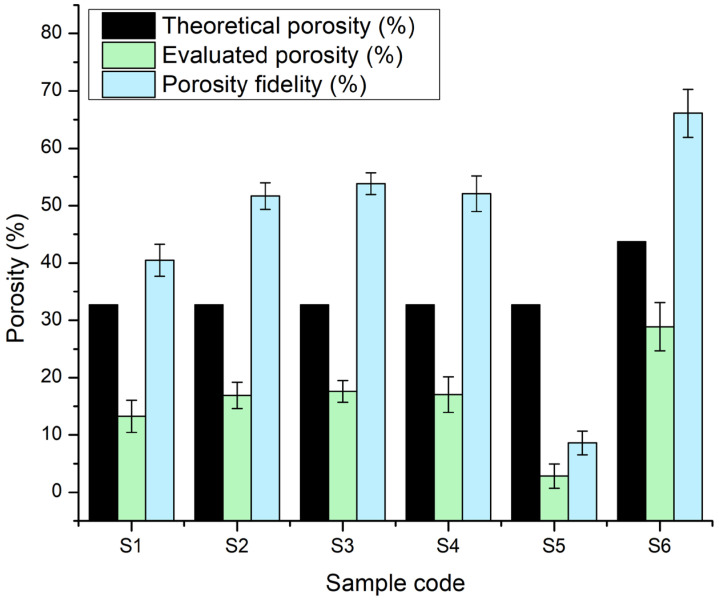
Comparative graph of the theoretical porosity calculated by the Bioscaffolds V2.0 software and the porosity evaluated through a Fiji image analysis for the 3D-printed scaffolds S1–S6.

The comparative graph of theoretical porosity (calculated using the BioScaffolds V2.0 application based on the printing parameters and designed geometry) and experimentally measured porosity (Figure 19) reveals how the actual behaviour of the hydrogels and hydrogel–CaP composites affected the scaffold architecture. The theoretical porosity remained constant at approximately ~32% for samples S1–S5 and increased to about 43% for sample S6, due to the use of a larger-diameter printing nozzle for this sample. However, the experimentally measured porosity was significantly lower for all the samples, indicating the partial blockage of the inter-filament spaces and layer fusion resulting from the filament swelling and hydrogel–CaP interactions.

Specifically, for sample S1, the experimental evaluation indicated approximately 13%, due to the non-Newtonian behaviour of the hydrogel, which caused increased filament swelling during printing and the subsequent layer fusion, thus reducing the designed pore spaces. For samples S2 and S3, containing 5% monetite and 5% brushite, respectively, the evaluated porosity was reduced to about 17–18%. The addition of the mineral phase stiffened the network and moderated some of the swelling effects, but sufficient filament expansion remained to significantly reduce the projected porosity.

Sample S4, without CaP and with a higher gelatine proportion, exhibited a measured porosity in the same range (~17%), due to the higher polymer network density conferred by the increased gelatine content. In contrast, sample S5, containing 3% monetite and a high gelatine proportion, recorded the lowest measured porosity (~2–3%), indicating nearly complete layer fusion, likely due to the combined influence of gelatine-associated filament swelling and the reinforcing effect of the monetite particles that created a dense network. For sample S6, which had the same CaP percentage as S5 (3%) but with brushite and the printing performed with a larger nozzle, the experimental-determined porosity was 28% compared to the theoretical value of 43%.

The porosity fidelity, defined as the percentage of the ratio between the evaluated and theoretical porosity, highlights these discrepancies quantitatively. Across all the samples, the porosity fidelity values were well below 100%, confirming that significant pore occlusion and morphological deviations occurred during and after printing. S6 exhibited the highest porosity fidelity (~66%), indicating that its printing strategy most closely preserved the originally designed architecture. In contrast, S5 had the lowest porosity fidelity (under 9%), revealing extensive pore loss. The moderate fidelity of S1–S4 (around 40–53%) reflects the persistent challenge of maintaining an open, interconnected porosity, especially in softer or highly swelling hydrogel systems.

#### 2.2.8. Swelling Degree

**Figure 20:**
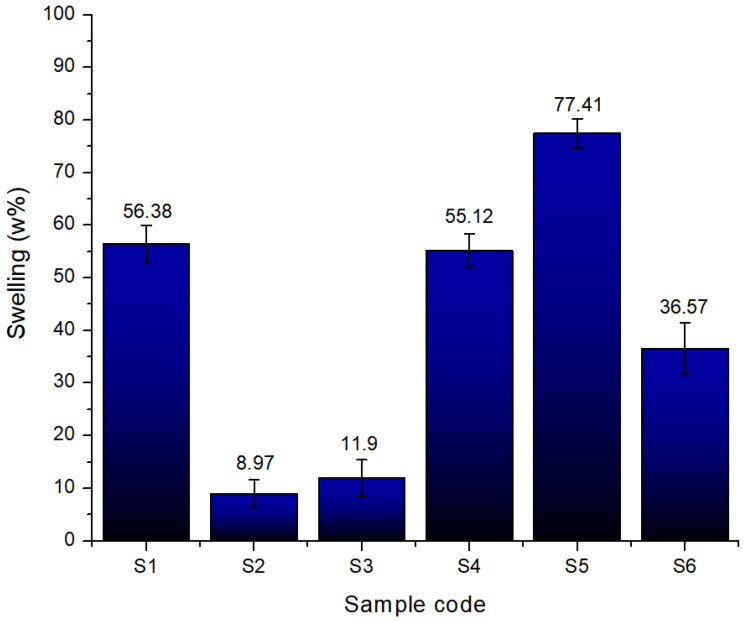
Sweeling degree of the 3D-printed scaffolds S1–S6.

The swelling rate, expressed as a percentage, reflects each sample’s capacity to absorb and retain fluid. The graph in Figure 20 clearly illustrates the influence of each component on the final volume. In the absence of a mineral phase, sample S1 (8% gelatine, 7% alginate, and 1% CMC) exhibited a high swelling rate of approximately 56%, indicating an open, flexible polymer network capable of expanding its intermolecular spaces to absorb fluid rapidly.

In contrast, the introduction of CaP significantly altered this behaviour: S2 (with 5% monetite) showed a low rate (~9%) due to network rigidification, while S3 (5% brushite) had a slightly higher swelling (~12%), reflecting different polymer–mineral interactions.

In the formulations with increased gelatine (12%) and lower alginate (5%), these trends are more pronounced. S4 swelled about 50%, slightly less than S1, due to a denser polymer network. Interestingly, S5 (3% monetite) showed a high swelling rate (~75%), suggesting that a moderate mineral content combined with a higher gelatine concentration allows for considerable expansion before the rigidity limits swelling. S6 swelled to ~36%, indicating that even at 3% the brushite imposed a much stronger constraint on network expansion compared to the monetite, reflecting how the nature of the phosphate influences the hydrophilic properties.

Overall, the swelling behaviour depends on both the mineral type and content, as well as the polymer composition. Scaffolds without minerals or with low monetite in gelatine-rich matrices are suited for applications needing high expansion, whereas those with brushite or a higher mineral content provide dimensional stability by limiting swelling.

#### 2.2.9. Degradation Rate

The degradation tests performed in phosphate-buffered saline (PBS) at 37 °C over 28 days demonstrated that the scaffold stability’s was strongly influenced by the interplay between the polymeric and inorganic phases. The hydrogel-only sample S1 (8% gelatine, 7% alginate, and 1% CMC) exhibited rapid degradation, losing more than 50% of its initial mass within the first week and surpassing 90% mass loss by day 28. In contrast, sample S4 (12% gelatine, 5% alginate, and 1% CMC) showed a more gradual degradation profile, with approximately 30% mass loss in the first 7 days and around 80% by day 28, resulting from the higher gelatine content.

The monetite-containing samples (S2 and S5) degraded more slowly, losing only 15–20% mass in the first week and about 60% by day 28, likely due to the monetite reinforcing the composite network. The brushite-containing samples (S3 and S6) exhibited intermediate degradation, with ~40% mass loss in the first week and ~80% by day 28. Despite brushite’s higher solubility, its interaction with the gelatine and alginate appeared to accelerate degradation after two weeks.

**Figure 21:**
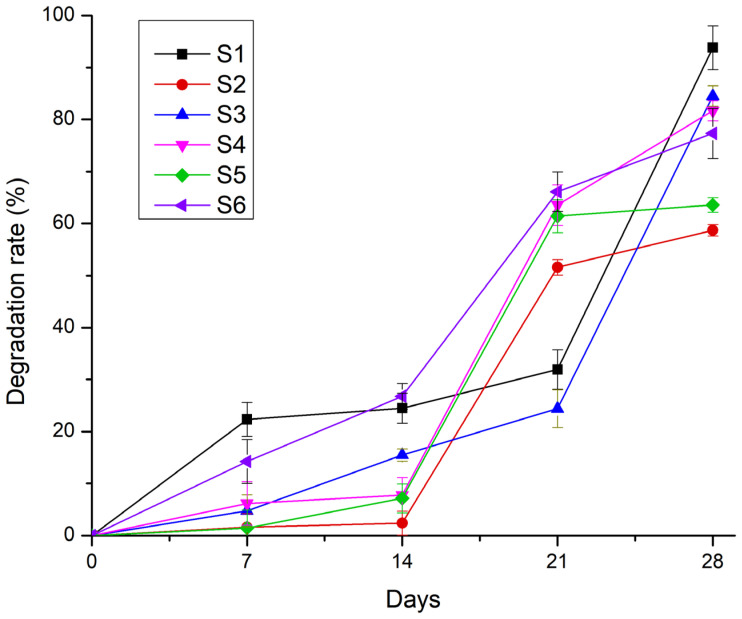
Degradation rate of the 3D-printed scaffolds S1–S6 after 7, 14, 21, and 28 days.

The degradation profile illustrated in Figure 21 highlights that the presence of a mineral phase delays the onset of rapid degradation, while the specific type of CaP modulates the long-term resorption rate of the scaffolds.

#### 2.2.10. Mechanical Properties

**Figure 22:**
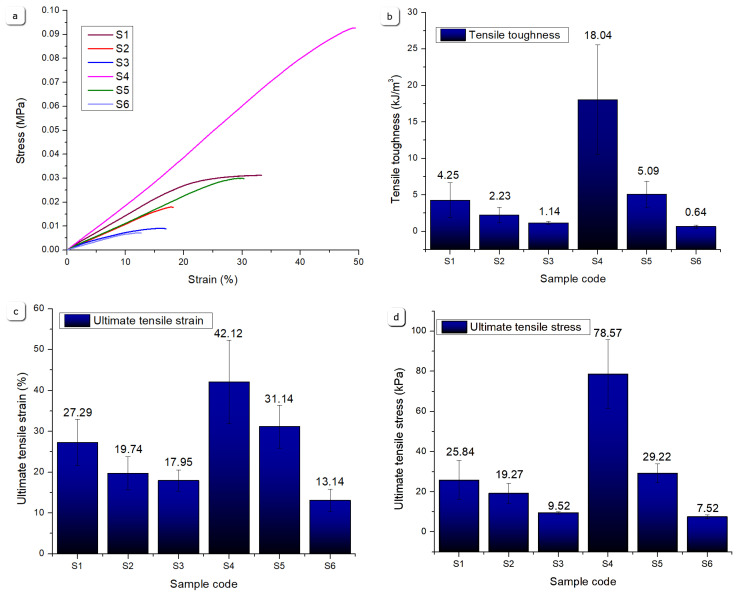
Comparative results of the tensile tests for S1–S6 samples: (**a**) strain as a function of stress, (**b**) tensile toughness, (**c**) ultimate tensile strain, (**d**) ultimate tensile stress values.

Figure 22 presents a comparative analysis of the uniaxial tensile test results, providing a structured framework for evaluating the mechanical properties of the printed scaffolds under tensile stress. The stress–strain comparisons highlight that Sample S4 exhibited superior mechanical performance, achieving the highest ultimate tensile stress among all the tested specimens. This enhanced performance can be attributed to its composition, containing 12% gelatine, 5% alginate, and 1% CMC without mineral reinforcement, resulting in a denser polymer network with strong intermolecular interactions and an increased crosslink density. The higher gelatine concentration contributed to the improved elasticity and tensile strength by providing a more cohesive matrix capable of sustaining a greater deformation and load.

Based on the tensile test results, Sample S4 demonstrated the most favourable mechanical characteristics, consistently surpassing the other samples in terms of stress–strain response. Its high tensile toughness suggests an improved capacity for energy absorption under tensile loading, indicative of a well-balanced polymeric structure.

Samples S5 and S1 also exhibited notable mechanical resilience, showing comparable tensile toughness values. Sample S5 contained 12% gelatine, 5% alginate, 1% CMC, and 3% monetite, where the incorporation of monetite particles reinforced the polymer matrix and restricted excessive deformation. This composite effect enhanced strength but slightly reduced the elasticity compared to S4. Sample S1, a hydrogel-only formulation (8% gelatine, 7% alginate, and 1% CMC), had a more flexible but less dense polymer network, affording reasonable tensile toughness but limited ultimate stress compared to S4, due to the lower crosslink density and absence of reinforcing minerals.

In contrast, Samples S2, S3, and S6 showed inferior performance across the tensile parameters. Sample S2 (8% gelatine, 7% alginate, 1% CMC, and 5% monetite) and S3 (8% gelatine, 7% alginate, 1% CMC, and 5% brushite) contained higher mineral contents but a lower gelatine concentration, resulting in a stiffer yet more brittle matrix prone to fracture under tensile stress. Sample S6 (12% gelatine, 5% alginate, 1% CMC, and 3% brushite), despite its higher gelatine, incorporated brushite, which interacted differently with the polymer matrix, likely creating localized stress concentrations that reduced the tensile toughness and strain capacity. The lower ultimate tensile strain and stress observed in Sample S6 reflect reduced structural integrity and mechanical robustness.

Overall, the tensile test results designate Sample S4 as the most mechanically robust under tensile loads, followed by the polymer–mineral composite S5, while the mineral-rich samples with either low gelatine or brushite exhibited diminished tensile resistance, due to the increased porosity generated by the incorporation of air bubbles in the preparation stage. These findings underscore the role of polymer composition—particularly the gelatine content—and mineral phase type and concentration in tuning the tensile performance.

**Figure 23:**
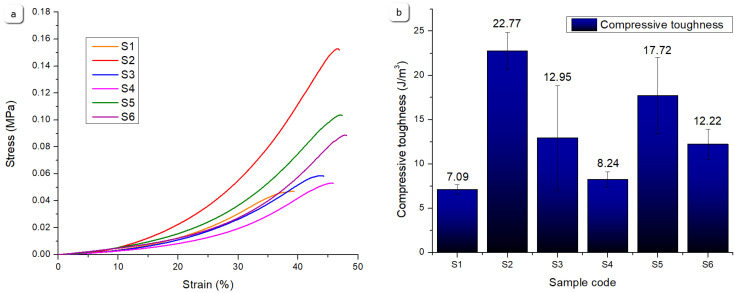

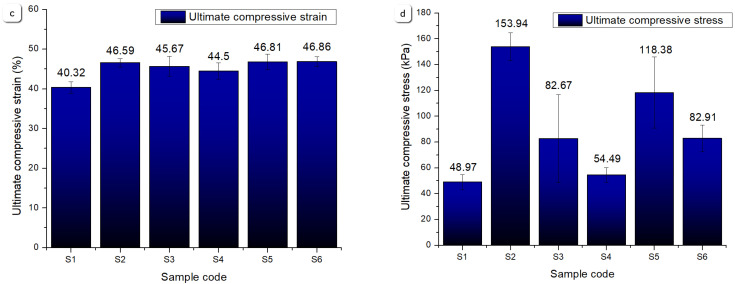
Comparative results of the uniaxial compression test for the S1–S6 samples: (**a**) strain as a function of stress, (**b**) compressive toughness, (**c**) ultimate compressive strain, (**d**) ultimate compressive stress values.

Figure 23 summarizes the compression test results, revealing notable variations in the mechanical resistance under compressive loading. Sample S2 demonstrated the highest compressive robustness, exhibiting superior compressive toughness and ultimate compressive stress at ~45% strain. This enhanced behaviour arises from its composite formulation of 8% gelatine, 7% alginate, 1% CMC, and 5% monetite, where the monetite acts as a reinforcing agent, stiffening the network and improving its load-bearing capacity. The moderate gelatine content allows for a balance between flexibility and strength under compression.

Sample S5 also showed a strong compressive performance, benefiting from a higher gelatine content (12%) combined with 3% monetite. A greater gelatine concentration contributes to the polymer network density enhancing the elasticity, while the monetite maintains reinforcement, yielding high compressive toughness and stress values.

Sample S6, with 3% brushite and the same polymer base as S5, displayed a lower ultimate compressive stress, possibly due to brushite’s different interaction with the polymer matrix, leading to reduced stiffness and earlier failure under compression.

Samples S1, S3, and S4 exhibited lower compressive resistance. Sample S1, without mineral addition and with a lower gelatine content, showed the lowest ultimate compressive stress and toughness, reflecting a looser polymer network vulnerable to deformation. Sample S3, despite its mineral content, had a low gelatine content and included brushite, resulting in a comparatively brittle structure. Sample S4, while outstanding in the tensile metrics, showed reduced compressive resistance, due to its polymer-only nature and higher elasticity, which allows for more deformation under compressive loads. These differences highlight how variations in the polymer content and mineral phase type govern compressive mechanics, enabling tailored scaffold design depending on the loading requirements, and are consistent with the literature data [40].

#### 2.2.11. LIVE/DEAD Viability Assay

**Figure 24:**
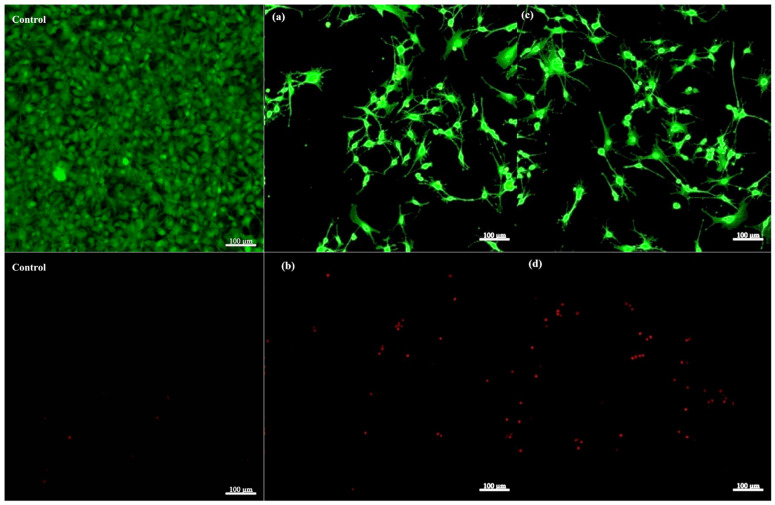
Confocal fluorescence microscopy images of the control substrate and S5 scaffold after LIVE/DEAD assay on human osteoblasts: (**a**,**c**) green fluorescence channel with viable cells, (**b**,**d**) red fluorescence channel with non-viable cells.

Figure 24 shows the results from the LIVE/DEAD fluorescence microscopy assay used to assess the viability of osteoblasts cultured on a 2D control flask substrate and the composite scaffold S5, which was selected due to its promising mechanical performance and favourable degradation, stability, and swelling profiles identified in the preliminary evaluations, making it a representative candidate for biocompatibility assessment. Two different regions from the same scaffold sample were studied to capture the spatial variability, accounting for potential heterogeneity in the cell distribution. Additionally, z-stacking was employed to visualize the three-dimensional arrangement of cells within the scaffold, allowing for an evaluation of viability both on the surface and deeper within the material.

Notably, the control group exhibited a higher density of clustered cells compared to the scaffold. This was primarily due to the 2D nature of the control substrate, where all the cells grew as a monolayer confined to the same focal plane. In contrast, the three-dimensional topography and porous architecture of the scaffold supported cell distribution throughout its volume, resulting in less apparent clustering in any single image plane. Moreover, the scaffold’s 3D structure promoted cellular extension and network formation—features absent in the 2D control group, where the osteoblasts lacked extended adhesion or cellular processes. This limited spreading and network formation is a known disadvantage of 2D culture systems, which fail to replicate the complex three-dimensional microenvironment. Furthermore, a small number of dead cells were observed in the 2D control group, where the rigid, flat surface could limit nutrient diffusion and alter the adhesion dynamics compared to the scaffold, contributing to localized cell death.

In the calcein AM channel images (Figure 24a,c), there is a high density of green fluorescent cells, indicating a predominantly viable osteoblast population on the scaffold, where the cells exhibit a well-spread morphology with extended processes, characteristic of healthy adherent osteoblasts on a supportive substrate, with cell-binding properties that may be enhanced through surface engineering.

Images (b, d) illustrate a small number of red fluorescent cells, suggesting a low proportion of non-viable (dead) osteoblasts, with one possible explanation for this cell death being represented by the insufficient removal of residual glutaraldehyde following the crosslinking step. Glutaraldehyde is known for its cytotoxicity, and inadequate washing after crosslinking can result in residual amounts within a scaffold, negatively affecting cell viability [41].

Overall, the LIVE/DEAD assay demonstrates that the composite scaffold supports good osteoblast viability, with a sizable proportion of cells alive and morphologically healthy, and minimal cell death observed across the tested surfaces, suggesting the scaffold has favourable cytocompatibility, making it suitable for bone tissue engineering applications.

## 3. Conclusions

This study investigated the development of composite systems fabricated by 3D printing, based on natural polymers (alginate, gelatine, and carboxymethylcellulose) and inorganic phases (monetite and brushite), aiming to evaluate their potential for bone tissue regeneration. Six distinct compositions (S1–S6) were formulated and characterized by varying gelatine and alginate concentrations, as well as mineral phase type and content. The samples were analysed morphologically, structurally, mechanically, and functionally.

The mechanical analyses revealed that the inclusion of mineral phases significantly enhanced the compressive and tensile strength. The compositions containing monetite (S2, S5) exhibited greater stiffness compared to those with brushite (S3, S6), confirming the stabilizing role of monetite. The mineral-free compositions (S1, S4) showed lower mechanical properties, but provided valuable insights into polymer behaviour without inorganic components. Increasing the gelatine content from 8 to 12% partially improved the mechanical strength and network cohesion, though not to the extent induced by a mineral phase presence.

The rheological analysis of this composite revealed that the shear sweep and flow curves closely resembled those of the hydrogel matrix without an inorganic phase. The minimal impact on the rheological response indicated that CaP incorporation did not significantly alter the viscoelastic properties of the material.

The swelling and degradation tests confirmed the superior stability in aqueous environments of the monetite-containing samples relative to the brushite-containing ones. The FTIR spectroscopy highlighted the presence and interaction of chemical components, particularly the formation of characteristic bonds between the polymers and calcium phosphates. The SEM investigation revealed significant morphological differences between the dried samples and those incubated for 28 days in simulated body fluid (SBF), with the latter showing extensive surface crystallization and well-developed crystals—indicative of bioactivity and osteoconductive potential.

The results demonstrate that polymer–mineral composite scaffolds can be effectively processed via 3D bioprinting, yielding constructs with tuneable properties depending on the composition. Compositions S2 (8% gelatine, 7% alginate, 1% CMC, and 5% monetite) and S5 (12% gelatine, 5% alginate, 1% CMC, and 3% monetite) stood out as the most balanced regarding the mechanical performance, wet stability, and printability, while all the formulations contributed to understanding polymer–mineral system behaviour under simulated physiological conditions.

The developed scaffolds represent a significant advancement in customizable bone graft materials, demonstrating superior mechanical properties and biocompatibility compared to single-phase systems. The incorporation of resorbable calcium phosphates not only enhanced the structural integrity but also promoted bioactivity through controlled mineral deposition. These findings establish a new paradigm for rational scaffold design, where the composition can be precisely tailored for specific clinical applications.

Future directions include refining the printing processes to achieve more complex geometries and controlled porosity, extending the study to in vitro cell culture assays assessing migration and proliferation, and also integrating osteoinductive or antibacterial agents into the matrix.

## 4. Materials and Methods

### 4.1. Materials

Sodium alginate (CAS 9005-38-3, Sigma-Aldrich), porcine gelatine (CAS 9000-70-8, Sigma-Aldrich), and carboxymethylcellulose (CMC, CAS 9004-32-4, DanidaCHEM, Bucharest, Romania) were obtained as biocompatible natural polymers. For calcium phosphate synthesis, calcium nitrate tetrahydrate (Ca(NO_3_)_2_·4H_2_O, 99.5% purity, Merck, Darmstadt, Germany), diammonium hydrogen phosphate ((NH_4_)_2_HPO_4_, Sigma-Aldrich), calcium carbonate (CaCO_3_, 99.5% purity, Merck), and phosphoric acid (H_3_PO_4_, 85% concentration, Sigma-Aldrich) were used as analytical-grade reagents. Calcium chloride (CaCl_2_, CAS 10043-52-4) for crosslinking and glutaraldehyde (0.5% solution, CAS 111-30-8) for stabilization were purchased from Sigma-Aldrich (St. Louis, MO, USA). Citric acid (3% solution) was used as additional crosslinking agent.

### 4.2. Synthesis of Calcium Phosphate Powders

#### 4.2.1. Synthesis of Monetite

**Figure 25:**
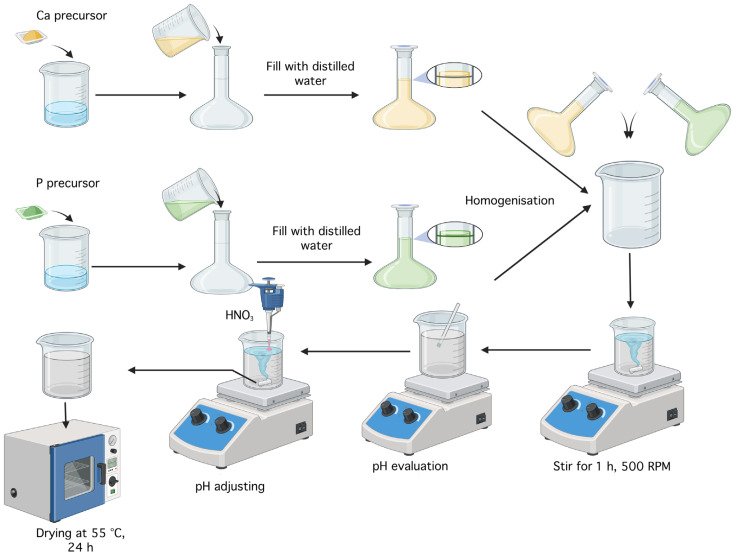
Schematic diagram of the monetite synthesis workflow (created with Biorender, https://www.biorender.com/).

Monetite was obtained using the coprecipitation method described in the scientific article by A.Y. Teterina et al. [42], involving the reaction between a 0.5 M solution of Ca(NO_3_)_2_·4H_2_O and a 0.5 M solution of (NH_4_)_2_HPO_4_, as shown in Figure 25. To prepare these solutions, 11.8075 g of the calcium precursor and 6.6025 g of the phosphorus precursor were each added to a Berzelius beaker along with 20 mL of distilled water. Each substance was then transferred into a 100 mL volumetric flask, brought to volume with distilled water, and homogenized. The two solutions were mixed in a Berzelius beaker, resulting in immediate precipitation, and the mixture was left on a hot plate without heating, under magnetic stirring at 500 rpm. After 1 h, the pH was checked and found to be 6; nitric acid (HNO_3_) was added dropwise until the pH reached 5. The resulting precipitate was washed with distilled water and centrifuged four times, each for 5 min at 6000 rpm The mixture was then left to dry in an oven at 55 °C for 24 h, yielding around 5.5 g of monetite powder.

#### 4.2.2. Synthesis of Brushite

**Figure 26:**
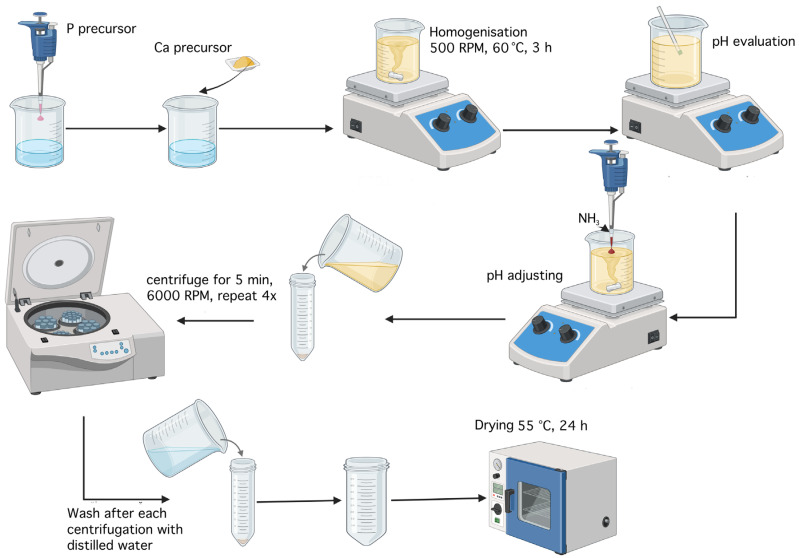
Schematic diagram of the brushite synthesis workflow (created with Biorender).

To obtain brushite, 200 mL of distilled water, 1.6014 g of CaCO_3_, and 695 µL of H_3_PO_4_ were used. Initially, the phosphorus precursor was added to the 200 mL of distilled water, followed by the addition of calcium precursor. The mixture was stirred magnetically at 500 rpm at a temperature of 60 °C. After 8 min, the formation of a precipitate was observed. After 3 h, the pH of the solution was measured. The initial pH was 4; however, according to the protocol described in the literature [43], the aim was to adjust it to pH 5. Ammonia was added dropwise until the desired value was reached. The resulting precipitate was washed with distilled water and centrifuged four times, each for 5 min at 6000 rpm. Subsequently, the product was dried at 55 °C for 24 h, yielding around 1.5 g of brushite powder. The synthesis steps are illustrated in Figure 26.

### 4.3. Synthesis of Hydrogel Compositions

The specific concentrations of gelatine (8–12%), alginate (5–7%), and CMC (1%) were selected based on optimization studies from the recent literature and our preliminary experiments on printability and mechanical enhancement, while calcium phosphate concentrations above 5% significantly compromise extrusion consistency and layer adhesion. Monetite was selected for its superior stability and controlled degradation compared to brushite, with slower dissolution rates under physiological conditions. Conversely, brushite provides rapid initial ion release for early osteoinduction and cellular activation [9].

**Figure 27:**
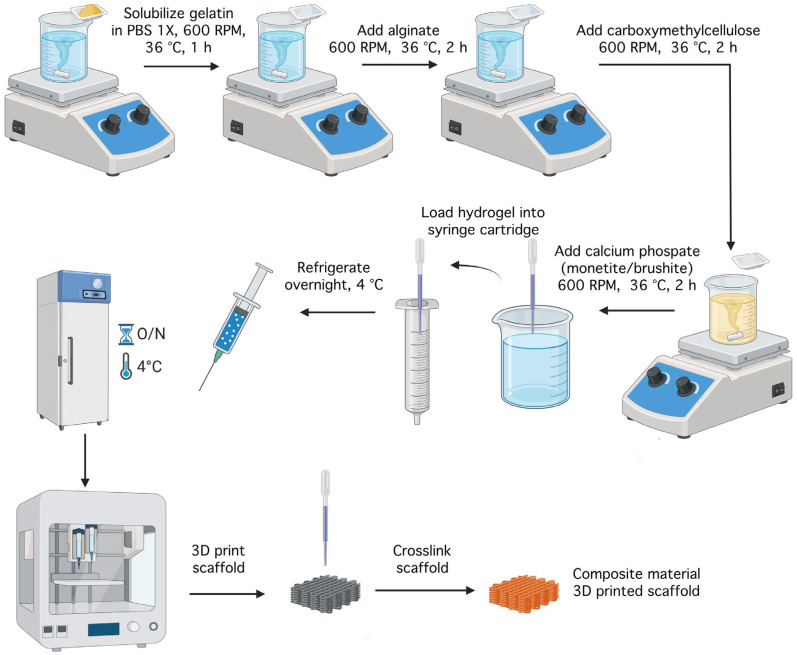
Schematic diagram of the scaffolds processing workflow (created with Biorender).

For the preparation of hydrogels with compositions as described in Table 1, 50 mL of PBS 1x solution was placed in a Berzelius beaker with a magnetic stirrer on a heated plate (DIAB MS-H280-Pro) at 600 rpm and 36 °C to prevent gelatine denaturation. The desired amounts of precursors were added, including sodium alginate, gelatine, and carboxymethylcellulose, as shown in Figure 27. Once the gelatine was completely dissolved, the alginate was added, followed by the CMC after homogenization, and the mixture was stirred for an additional 2–3 h to ensure complete incorporation.

The resulting hydrogels were divided into three equal parts using syringes, and the calcium phosphates were added, maintaining the temperature and magnetic stirring until complete incorporation was achieved. Thus, the six compositions presented in Table 1 were obtained, which were then loaded into special cartridges for bioprinting and placed in a freezer for 18 h at 4 °C to facilitate gelation.

### 4.4. Three-Dimensional Printing Process

**Figure 28:**
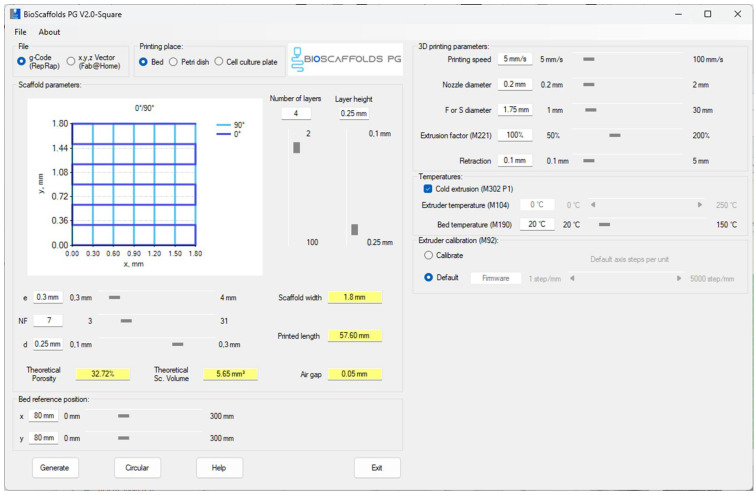
Screenshot from Bioscaffolds V2.0 software illustrating the parameters used for GCode generation.

The compositions were printed using a CELLINK INKREDIBLE+ extrusion-based 3D bioprinter, (Gothenburg, Sweden). GCode files were generated with Bioscaffolds V2.0 software (Figure 28) [44]. The optimal printing parameters for the obtained bioink are detailed in Table 1. Extrusion was performed using a 22G conical nozzle for compositions S1–S5, and using a 25G conical nozzle for composition S6. Square-grid scaffolds measuring 1.80 mm × 1.80 mm were printed in 90 mm Petri dishes. Following printing, the scaffolds underwent dual crosslinking: first with a 1.66% CaCl_2_ solution to rapidly stabilize the alginate and form a primary network supported by reversible ionic bonds; then with a 0.5% glutaraldehyde and 3% citric acid solution to consolidate the gelatine and carboxymethylcellulose, thereby enhancing the mechanical strength of the assembly [45]. Each crosslinking step lasted 15 min.

### 4.5. Characterization Methods

#### 4.5.1. X-Ray Diffraction

X-ray diffraction (XRD) serves as a qualitative and quantitative method to identify the present phases; crystal alignment; and structural aspects, including the average crystallite size, crystallinity degree, and crystal defects. An XRD analysis was conducted using a Shimadzu XRD-6000 instrument (Shimadzu, Kyoto, Japan) with a Bragg–Brentano configuration, equipped with a copper anode X-ray tube (Cu K*α* wavelength *λ* = 1.541874 Å). The diffraction patterns were collected over a 2*θ* range of 5–60°, with a step size of 0.02° and 100 s/step.

#### 4.5.2. Fourier Transform Infrared Spectroscopy

Fourier transform infrared spectroscopy (FTIR) is commonly used for physicochemical characterization of organic materials. Each absorption band corresponds to vibrations of chemical bonds between atoms; if a bond is characteristic of a component, it serves as an indicator of it. The powder measurements were performed using a Thermo Scientific Nicolet iS50 spectrometer (Thermo Fisher Scientific, Waltham, MA, USA) at room temperature with an attenuated total reflectance (ATR) module, collecting 32 scans between 4000 and 400 cm^−1^ at a 4 cm^−1^ resolution. FTIR spectra of 3D-printed scaffolds were acquired in the 4000–500 cm^−1^ range using a Jasco FT-IR 42 ATR spectrometer (Jasco Global, Tokyo, Japan). The samples were dried and placed on the ATR accessory before measurement.

#### 4.5.3. Scanning Electron Microscopy and Energy-Dispersive X-Ray Spectroscopy

Scanning electron microscopy (SEM) is considered a non-invasive technique for examining the morphological features of materials. It offers spatial resolutions between 50 and 100 nm and can magnify samples from 20 up to 200,000 times their original size. The SEM micrographs revealed details about the particle morphology, size distribution, and agglomeration tendencies in the calcined powders. These examinations were performed using a FEI Quanta Inspect F50 scanning electron microscope (Thermo Fisher Scientific, Waltham, MA, USA), which allows for variable magnification. Prior to analysis, both the powders and scaffold samples were coated with a thin gold layer via sputter coating to improve conductivity.

Energy-dispersive X-ray spectroscopy (EDX or EDS) enables an elemental composition analysis by detecting the characteristic X-rays emitted when an electron beam interacts with a sample. Since each element has a unique energy spectrum, this technique allows for identification of constituent elements. Moreover, the intensity of spectral peaks provides information on the relative abundance of these elements within a sample. EDX is generally integrated within SEM systems.

#### 4.5.4. Uniaxial Tensile Test

**Figure 29:**
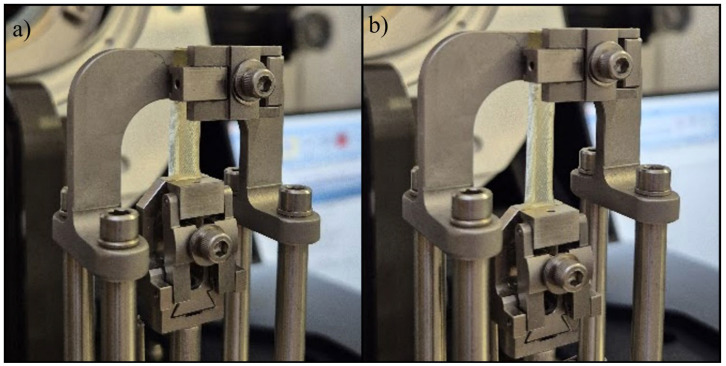
Aspect of the scaffold sample S4: (**a**) before and after (**b**) tensile test setup.

Uniaxial tensile tests were conducted to evaluate the mechanical properties of the printed scaffolds using a Discovery 850 DMA (TA Instruments, New Castle, DE, USA) equipped with a film clamp setup for uniaxial tensile testing (Figure 29a,b). The tests were performed in rate control mode with a strain ramp applied to rectangular specimens measuring approximately 40 mm × 8 mm × 2 mm. Testing was carried out at a constant displacement rate of 5 mm/min, under controlled environmental conditions of 25 °C, with a preload force of 0.01 N and a sampling rate of 10 points/s. All the tensile evaluations adhered to ASTM D3039 [46] standards. For each type of sample, five specimens were tested under identical conditions, with the average values reported. To ensure clarity of comparative analysis, only a single representative curve from each set—corresponding to the parameters nearest to the average values—was included in the graphical representations within Section 2.

#### 4.5.5. Uniaxial Compression Test

**Figure 30:**
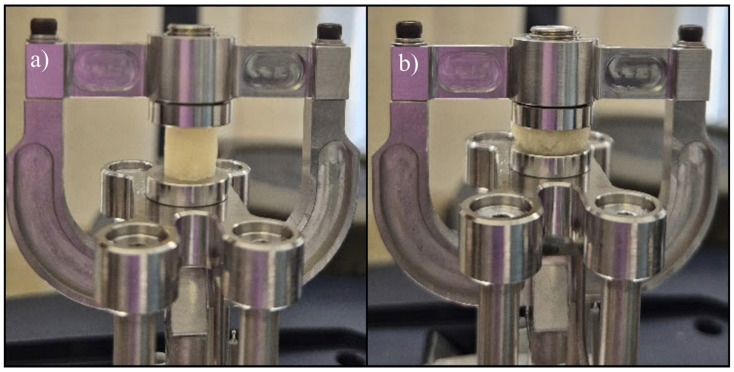
Aspect of the scaffold sample S5: (**a**) before and after (**b**) compression test setup.

Uniaxial tensile compression tests were conducted to assess the compressive resistance of the printed scaffolds using the same instrument, a Discovery 850 DMA (TA Instruments, New Castle, DE, USA), now equipped with a compression clamp setup for uniaxial compression testing (Figure 30a,b). The experiments were performed in rate control mode, applying a strain ramp to cylindrical specimens with approximate dimensions of *ϕ* = 9 mm and *h* ≈ 9 mm. Testing was executed at a constant ramp rate of 5 mm/min under controlled environmental conditions of 25 °C, with a preload force of 0.01 N and a sampling rate of 10 points/s. For each sample type, five specimens were subjected to testing under identical experimental conditions, and the corresponding average values were recorded. To enhance clarity of comparative plots, only a single representative curve from each set, corresponding to the parameters most closely aligned with the average values, was incorporated into the graphical representations within Section 2.

#### 4.5.6. Rheological Evaluation

The rheological characterization of viscoelastic materials requires a systematic approach involving multiple testing methodologies to fully understand their mechanical behaviour using oscillatory shear testing, specifically amplitude sweeps and frequency sweeps and shear testing. Prior to the measurements, each sample (S4 and S5) was pre-conditioned at the testing temperature for 2 min. The rheological analyses were conducted using an Anton Paar rheometer (Anton Paar GmbH, Graz, Austria) equipped with a 25 mm diameter parallel-plate geometry. Hydrogel sample S4 was loaded onto the lower plate, with the gap set to 0.5 mm. Composite sample S5 was also evaluated, with negligible differences observed compared to the pristine hydrogel. The amplitude sweep tests were performed at a constant frequency of *ω* = 1 s^−1^ to determine the linear viscoelastic region (LVR) for both samples. The LVR represents the strain range where the material structure remains intact, and the viscoelastic moduli are independent of strain amplitude. The frequency sweeps were conducted at a fixed strain amplitude of *γ* = 0.01, well within the previously determined LVR. This approach allowed for the non-destructive characterization of the materials’ time-dependent properties.

The samples were subsequently characterized using a steady shear test, which measured the variation in the viscosity as a function of shear rate. All the measurements were carried out at 25 ± 0.1 °C, maintained by a temperature-controlled Peltier system.

#### 4.5.7. Filament Collapse Testing

The filament collapse testing employed five central pillars (2 × 10 × 6 mm^3^) and two end pillars (5 × 10 × 6 mm^3^) spaced at known distances of 1, 2, 3, 4, 5, and 6 mm, as described in [30]. Printing was performed at 5 mm/s using a 22G nozzle for compositions S1–S5 and a 25G nozzle for S6. The printing temperature was maintained at 25 °C. To avoid unfavourable deformation, the printed filament was immediately imaged with a high-resolution camera after suspension.

#### 4.5.8. Three-Dimensional Printing Accuracy

Printing accuracy of the 3D-printed scaffolds was evaluated via optical microscopy. To quantitatively assess the printing accuracy, the thickness of gel filaments within the scaffolds was analysed by comparing the measured filament dimensions to the nozzle diameter. The filament thickness was recorded using the Measure Function in Fiji. Triplicate specimens for each sample category were tested under uniform conditions, and the reported values represent their averages.

#### 4.5.9. Porosity Evaluation

**Figure 31:**
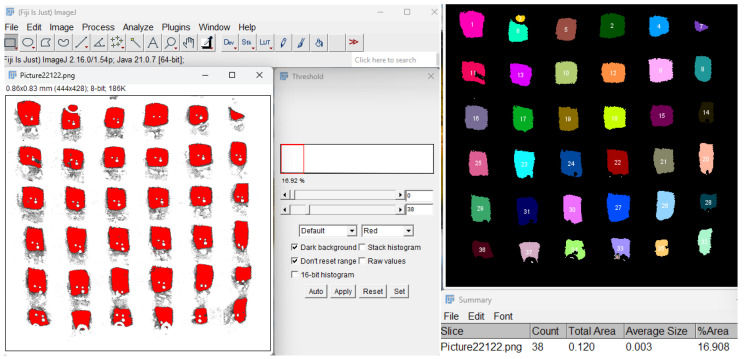
Porosity evaluation using Fiji software (1.54p version) and the Particle Analyzer function on a thresholded 8-bit image of S2.

Porosity of the 3D-printed scaffolds was assessed using Fiji software [47], as illustrated in Figure 31. Digital images of the scaffolds were first processed using the Threshold function to segment the solid and void regions by setting a specific contrast range. This step enabled differentiation between the polymer matrix and porous spaces by converting the image to grayscale and then to a binary format. Subsequently, the Particle Analyzer tool quantified the porosity parameters by counting and measuring the individual pore areas and their distribution within the scaffold. For each type of sample, three specimens were tested under identical conditions. Additionally, the porosity fidelity of each scaffold construct was quantified to evaluate how accurately the printed architecture matched the theoretical design.

The porosity fidelity was calculated using the following Formula (1):

(1) $$Porosity\;fidelity\left(\%\right)=\frac{Evaluated\;porosity}{Theoretical\;porosity}\times 100$$

#### 4.5.10. Swelling Degree

The scaffolds were initially weighed dry, then immersed in 1× PBS solution. After 4 h, ensuring complete PBS absorption, the scaffolds were weighed again. The measurements were performed on three identical samples for each hydrogel composition. The liquid retention capacity of each construct was quantified using the following Equation (2):

(2) $$Swelling\;rate\left(\%\right)=\frac{W_{wet}-W_{dry}}{W_{dry}}\times 100$$

where $W_{wet}$ is the scaffold weight after 1 × PBS immersion, and $W_{dry}$ is the scaffold weight before immersion.

#### 4.5.11. Degradation Rate

To evaluate the degradation profiles, both the unmodified scaffolds (gelatine and alginate only) and composite scaffolds containing monetite or brushite were immersed in 3 mL of 1× PBS at 37.1 °C. The scaffold weights were recorded at regular intervals (1, 7, 14, 21, and 28 days) to monitor their degradation over time. Each sample type was tested in triplicate under consistent conditions.

#### 4.5.12. In Vitro Mineralization

To evaluate the in vitro mineralization and apatite formation, the composite scaffolds were placed in Petri dishes and immersed in 3 mL of 1 × simulated body fluid (SBF) [48], prepared according to the protocol described by Kokubo et al. [49]. The samples were then incubated in a temperature-controlled incubator at 37.1 °C. After an incubation period of 28 days, the scaffolds were examined using scanning electron microscopy (SEM) to assess the biological response.

#### 4.5.13. LIVE/DEAD Viability Assay

Human foetal osteoblastic cells (hFOB 1.19) [50] were maintained under standard culture conditions in a humidified incubator containing 95% air and 5% CO_2_ at 37 °C. The cells were grown in Dulbecco’s Modified Eagle’s Medium (DMEM) containing 10% foetal calf serum and 1% penicillin–streptomycin supplementation. Upon reaching confluence, the cells underwent trypsinization and were subsequently counted. The scaffold materials were sterilized using UV-C irradiation for 20 min per side before being immersed in the culture medium. Each scaffold received 2 × 10^5^ cells in 2000 μL of DMEM per well, followed by a 48 h incubation period at 37 °C. A cell viability assessment was conducted using a LIVE/DEAD assay kit following the manufacturer’s protocol (Invitrogen, Thermo Fisher Scientific, Waltham, MA, USA) [51]. The procedure began with the removal of growth medium from the scaffold samples, followed by gentle PBS washing to eliminate loose cells and debris. A working staining solution was freshly prepared by mixing equal volumes of diluted calcein AM (2 µM) and propidium iodide (2 µM) solutions. The samples were treated with this staining solution and incubated in darkness at room temperature for 30–60 min, allowing sufficient time for dye penetration and binding to their respective intracellular targets. After the incubation period, the samples underwent PBS rinsing to remove the excess, unbound staining reagents. Fluorescence microscopy was performed using a Zeiss LSM system equipped with appropriate filter sets for green and red fluorescence detection. Image acquisition and a qualitative analysis was accomplished using ZEN Studio 1.6.1 image analysis software to determine the viability (green fluorescence) of the cells and the non-viable osteoblasts (red fluorescence).

## Figures and Tables

**Table 1:** Three-dimensional-printed scaffolds compositions, printing parameters, and crosslinking conditions.

Sample	Gelatine (%)	Alginate (%)	CMC (%)	Monetite (%)	Brushite (%)	Printing Pressure (kPa)	Printing Speed (mm/s)	Nozzle Diameter	Crosslinking
S1	8	7	1	0	0	220	5	25G (260 µm)	1.66% CaCl_2_ 0.5% glutaraldehyde 3% citric acid–15 min
S2	8	7	1	5	0	263			
S3	8	7	1	0	5	195			
S4	12	5	1	0	0	283			
S5	12	5	1	3	0	274			
S6	12	5	1	0	3	231		22G (400 µm)	

## Data Availability

The data is contained within the article or Supplementary Materials.

## Supplementary Materials

The following supporting information can be downloaded at: https://www.mdpi.com/article/10.3390/gels11080665/s1.

## Abbreviations

The following abbreviations are used in this manuscript:

CaP	Calcium Phosphate
Alg	Alginate
Gel	Gelatine
CMC	Carboxymethylcellulose
FTIR	Fourier Transform Infrared Spectroscopy
SEM	Scanning Electron Microscopy
EDS	Energy-dispersive X-ray Spectroscopy
PBS	Phosphate-buffered Saline
SBF	Simulated Body Fluid
hFOB 1.19	Human Foetal Osteoblastic Cells
DMEM	Dulbecco’s Modified Eagle’s Medium
